# Eye, hepatobiliary, and renal disorders of erlotinib in patients with non-small-cell lung cancer: A meta-analysis

**DOI:** 10.1371/journal.pone.0234818

**Published:** 2020-07-14

**Authors:** Hye Duck Choi, Min Jung Chang

**Affiliations:** 1 College of Pharmacy, Yeungnam University, Gyeongsangbuk-do, Republic of Korea; 2 College of Pharmacy and Yonsei Institute of Pharmaceutical Sciences, Yonsei University, Incheon, Republic of Korea; 3 Department of Pharmaceutical Medicine and Regulatory Sciences, Colleges of Medicine and Pharmacy, Yonsei University, Incheon, Republic of Korea; Istituto di Ricovero e Cura a Carattere Scientifico Centro di Riferimento Oncologico della Basilicata, ITALY

## Abstract

**Background:**

Erlotinib is an epidermal growth factor receptor (EGFR) tyrosine kinase inhibitors used to treat EGFR mutation positive non-small-cell lung cancer (NSCLC). Skin rash and diarrhea are well-known and common adverse events in patients receiving erlotinib, whereas other adverse events, including eye, liver, or renal disorders have not been evaluated adequately. This meta-analysis aimed to evaluate the ocular, hepatobiliary, and renal toxicities of erlotinib in patients with NSCLC cancers.

**Methods:**

In total, sixty studies were assessed, and the results of the included studies were quantitatively integrated using meta-analysis. The incidence of ocular, hepatobiliary (alanine aminotransferase [ALT] and bilirubin elevations; other hepatic adverse events), and renal adverse events were estimated. Additionally, the erlotinib-treated groups and the control groups (placebo or other treatment) were compared with respect to ocular disorders and ALT elevation. The study protocol has been registered in the International Prospective Register for Systematic Reviews (PROSPERO) CRD42018093758.

**Results:**

The overall incidence of ocular disorders was 3.30% (95% confidence interval [CI] 2.20%–5.00%). The incidence of ALT elevation, bilirubin elevation, and other hepatobiliary disorders was 6.40% (95% CI 3.90%–10.4%), 3.80% (95% CI 2.30%–6.10%), and 1.00% (95% 0.60%–1.80%), respectively. The incidence of renal disorder was 3.10% (95% CI 1.90%–5.00%). The risk of ocular toxicity in the erlotinib treatment group was significantly increased (risk ratio = 2.91; 95% CI 1.70–4.98) compared to that in the control group. ALT elevation was not significantly different between the two groups.

**Conclusion:**

Based on the results, careful monitoring of ocular toxicity in patients receiving erlotinib should be recommended and closer monitoring of hepatic toxicity should be also recommended in patients with liver-related risk factors.

## Background

Many molecular genetic alterations are involved in the pathogenesis and progression of lung cancer. The epidermal growth factor receptor (EGFR) mutation is a genetic alteration and frequently observed in patients with non-small-cell lung cancer (NSCLC) [1, 2]. Therefore, erlotinib and other EGFR tyrosine kinase inhibitors (TKIs) such as gefitinib, afatinib, and osimertinib are the first-line therapies to treat EGFR mutation positive NSCLC in stage IIIB and IV [3].

The clinically important role and effectiveness of erlotinib have certainly been reported in lots of studies and published research. The information about its safety is also generally reported and well-known. Skin rash and diarrhea are the most common adverse events of erlotinib and the event rates were reported as 49.2% for skin rash and 20.3% for diarrhea in the SATURN study [4]. Dose reductions or delays due to these adverse effects may be required, but the first-line therapy remains erlotinib followed with monitoring and appropriate supportive care in many cases. That is, erlotinib-related skin rash and diarrhea have been evaluated carefully based on the clinical application and studies and how to manage about the adverse events are also well established.

Whereas, other erlotinib-related adverse events, such as eye, liver, or renal toxicities, have been considered less extensively, despite the fact that these risks have been reported continuously since the initial clinical trials of erlotinib [5, 6]. These adverse events occur less frequently and most of them are mild to moderate. But the event rates have a limitation in that they were reported individually as a unit of each study. Indeed, many studies have reported on the adverse events of erlotinib, but the results were inconsistent among studies. Shepherd et al. reported that an eye disorder occurred in 28 patients, but hepatic or renal events were not observed in a trial involving 485 participants [5]. In contrast, the liver-related event rate was 38.4% in a trial involving 276 participants and no ocular or renal disorders were reported [7]. This shows that, to date, there are no clear conclusions regarding the association of the aforementioned toxicity-related adverse events with erlotinib.

The aim of our study was to evaluate the eye, hepatobiliary, and renal disorders of erlotinib in patients with NSCLC and to integrate quantitatively the results through conducting a meta-analysis.

## Methods

### Search strategy and study selection

The MEDLINE (OVID and PubMed) and Cochrane Library were accessed for the literature searching in this meta-analysis. The following PubMed MeSH terms and related text terms were used: “erlotinib”, “cancer”, “neoplasm”, “carcinoma”, “clinical trials”, and “randomized clinical trials”. Searching for the bibliographies of all relevant articles were also performed. There was no publication limitation. The search was completed on 13 July 2018.

The review process for selecting article were conducted by same methods with our previously reported studied [8]. The inclusion criteria were described as below:

1. Phase II, III, and IV trials in patients with NSCLC
2. Participants who received daily erlotinib treatment
3. Inclusion of the reported adverse events or toxicity related data

The study protocol for this meta-analysis has been registered in the International Prospective Register for Systematic Reviews (PROSPERO) CRD42018093758 on May 30, 2018.

### Data extraction and quality assessment

The following data were extracted from each included study: the first author’s surname, publication year, study design, number of participants, type of cancer, treatments (dose regimen and periods), and toxicity related data.

The methodological quality of each study was evaluated by two authors according to the Jadad scale, which is using for the randomized controlled trials [9]. The scale evaluates as total 5 points about a description of the randomization, the appropriateness of the randomization method, a description of double blinding, the appropriateness of the double-blinding method, and a description of withdrawals and dropouts. Scores higher than 3 were considered high quality. Any discrepancies between the two authors were resolved by discussion.

### Statistical analysis

The end point for this meta-analysis was the incidence of eye, hepatobiliary, and renal adverse events following monotherapy with erlotinib for NSCLC. As a sub-group analysis, the incidence of eye and hepatic disorders in the erlotinib treatment group were compared with the values in the control group with placebo or cytotoxic chemotherapy. To evaluate the heterogeneity of the included studies, the *χ*^2^ test with *Q* statistics and quantified using *I*^2^ measures were applied [10]. A fixed-effects model (Mantel–Haenszel method) or a random-effects model (DerSimonian–Laird method) was applied in the calculations based on the result of heterogeneity test in each analysis [11, 12].

Sensitivity analysis was conducted to improve the reliability of meta-analysis. The meta-analytic calculations were repeatedly performed after each study was excluded in turn. To examine potential publication bias, the Begg’s test and Egger’s test [13, 14] were applied. All statistical analysis and calculations were performed using the Comprehensive Meta-Analysis software, version 2 (CMA 26526; Biostat, Englewood, NJ USA). All statistical tests were two-sided and *P-value* < 0.05 was considered to indicate statistical significance.

## Results

### Study quality and characteristics

In total, 1,511 articles were identified in the literature search. After the removal of duplicates, the titles and abstracts of 1,148 articles were screened. Of these, 934 articles were excluded, and the full texts of the remaining 214 articles were assessed for eligibility. A further 154 articles were excluded because of insufficient data, overlapped data, or because they were unsuitable based on the inclusion criteria. The remaining 60 studies were included in this meta-analysis. The study selection process was performed according to the Preferred Reporting Items for Systematic Reviews and Meta-Analysis (PRISMA) guidelines (available on S1 Fig) [15].

The characteristics of the 60 studies are listed in Table 1. Patients with stage IIIB and IV NSCLC were enrolled in the included studies and only one study was performed in patients with IIB and IIIA NSCLC [58]. Erlotinib at 150 mg daily was administered in the all included studies. Using the Jadad score, 37 studies were classified as low quality (a score of 2 and or less), whereas 23 studies were classified as high quality (a score of 3 or greater) (Table 1).

**Table 1:** General characteristics of the included studies.

Study	Study design	No. of participants		Adverse events				Jadad score
			Eye disorder	ALT increase	Bilirubin increase	Other hepatic disorder	Renal disorder	
Shepherd, 2005 [5]	Phase III	485	28	0	0	0	0	4
Arrieta, 2008 [16]	Phase II	150	0	0	0	0	0	1
Felip, 2008 [17]	Phase II	73	3	4	0	0	0	1
Hesketh, 2008 [18]	Phase II	76	0	0	0	0	1	1
Kubota, 2008 [19]	Phase II	62	0	15	15	0	0	1
Lee, 2008 [20]	Phase II	23	0	0	1	0	0	1
Lilenbaum, 2008 [21]	Phase II	52	0	1	0	0	0	3
Akerley, 2009 [22]	Phase II	40	0	0	3	0	0	1
Reck, 2010 [23]	Phase IV	6580	71	23	32	0	0	1
Rossi, 2010 [24]	Phase II	30	3	0	0	1	0	1
Stathopoulos, 2010 [25]	Phase II	54	0	0	0	0	0	1
Takahashi, 2010 [26]	Phase II	46	0	12	13	0	0	1
Yoshioka, 2010 [27]	Phase II	30	0	0	0	9	0	1
Choi, 2011 [28]	Phase II	75	0	2	0	0	0	1
Lee, 2011 [29]	Phase II	24	0	6	0	0	0	1
Matsuura, 2011 [30]	Phase II	20	0	0	0	0	0	1
Mita, 2011 [31]	Phase II	42	0	0	0	0	0	1
Natale, 2011 [32]	Phase III	614	0	0	0	0	0	4
Ramalingam, 2011 [33]	Phase II	57	0	0	0	0	0	4
Sequist, 2011 [34]	Phase II	83	0	1	0	0	5	4
Zhou, 2011 [35]	Phase III	83	NR	31	0	0	NR	3
Ciuleanu, 2012 [36]	Phase III	196	9	NR	NR	NR	NR	3
Kobayashi, 2012 [37]	Phase II	31	0	5	0	0	0	1
Lee, 2012 [38]	Phase III	334	3	NR	NR	NR	NR	4
Pérol, 2012 [39]	Phase III	155	0	0	0	0	8	3
Rosell, 2012 [40]	Phase III	84	NR	5	0	0	NR	3
Scagliotti, 2012 [41]	Phase III	477	NR	157	124	0	121	5
Schaake, 2012 [42]	Phase II	60	9	NR	NR	NR	NR	1
Witta, 2012 [43]	Phase II	63	0	0	0	0	0	4
Wu, 2012 [44]	Phase III	59	0	1	0	0	0	5
Goto, 2013 [45]	Phase II	103	NR	34	26	0	NR	1
Goren, 2013 [46]	Phase II	64	0	0	0	0	0	5
Wu, 2013 [47]	Phase II	48	3	4	8	0	NR	1
Yamada, 2013 [48]	Phase II	26	0	10	13	0	13	1
Brahmer, 2014 [49]	Phase II	135	9	13	21	0	0	1
Gemma, 2014 [50]	Phase IV	9909	331	NR	NR	NR	NR	1
Gitilitz, 2014 [51]	Phase II	42	NR	NR	NR	NR	0	4
Horiike, 2014 [52]	Phase II	50	0	9	18	0	10	1
Kawaguchi, 2014 [53]	Phase III	150	NR	39	0	0	NR	3
Matsumoto, 2014 [54]	Phase II	46	0	6	0	0	0	1
Morise, 2014 [55]	Phase II	53	3	14	0	0	0	1
Seto, 2014 [56]	Phase II	77	10	0	0	39	4	2
Yoshioka, 2014 [57]	Phase IV	477	NR	157	124	0	NR	1
Kelly, 2015 [58]	Phase III	611	61	NR	NR	NR	NR	4
Minemura, 2015 [59]	Phase II	16	0	3	0	0	2	1
Reckamp, 2015 [60]	Phase III	53	NR	27	6	0	8	5
Wu, 2015 [61]	Phase III	110	NR	13	11	0	NR	3
Yamada, 2015 [62]	Phase II	18	NR	3	7	0	NR	1
De Grève, 2016 [63]	Phase II	46	21	NR	NR	NR	NR	1
Lara, 2016 [64]	Phase II	32	NR	NR	NR	NR	8	1
Neal, 2016 [65]	Phase II	40	5	5	6	0	2	1
Papadimitrakopoulou, 2016 [66]	Phase II	22	NR	5	0	0	NR	3
Park, 2016 [67]	Phase II	207	37	26	14	0	NR	1
Urata, 2016 [7]	Phase III	276	NR	106	105	0	NR	1
Yamada, 2016 [68]	Phase II	40	0	16	16	0	16	1
Ciuleanu, 2017 [69]	Phase II	101	NR	5	1	0	18	5
Ikezawa, 2017 [70]	Phase II	19	NR	2	3	0	4	3
Leighl, 2017 [71]	Phase II	44	5	1	0	0	0	5
Miyawaki, 2017 [72]	Phase II	38	0	2	3	0	4	1
Yang, 2017 [73]	Phase III	128	NR	6	10	0	0	1

Abbreviations: ALT, alanine aminotransferase; NR, not reported.

### Incidence and relative risk of erlotinib-induced eye disorders

Forty-four of the 60 studies were assessed for the incidence of eye disorders in 20,964 participants treated with erlotinib (Table 1). In total, 611 eye-related adverse events of any grades were reported, and thirty three of the 611 adverse events were classified as grade 3–4. The incidence of eye disorders in each study was between 0.00% and 45.7%. The index of eye disorders mainly included dry eye, conjunctivitis, blurry vision, and other ocular adverse events.

The overall incidence (event rate) of eye disorders for any grade was 3.30% (95% confidence interval [CI] 2.20%–5.00%) using the random-effects model (Table 2).

**Table 2:** Incidence (event rate) of ocular, hepatobiliary, and renal disorders, test of heterogeneity and publication bias.

Types of disorder	Incidence (95% CI)		Heterogeneity			Publication bias	
	Fixed-effect model	Random-effect model	*Q v*alue	*P v*alue	*I*^2^	*P* value (Begg’s)	*P* value (Egger’s)
*Eye disorders*							
Any grade	0.041 (0.038–0.044)	0.033 (0.022–0.050)	471.0	0.000	90.87	0.723	0.954
3–4 grade	0.004 (0.003–0.005)	0.006 (0.004–0.008)	51.27	0.181	16.13	0.000	0.000
*Hepatobiliary disorders*							
ALT elevations (any grade)	0.113 (0.106–0.122)	0.064 (0.039–0.104)	1679	0.000	96.96	0.000	0.598
Bilirubin elevations (any grade)	0.157 (0.144–0.172)	0.038 (0.023–0.061)	843.7	0.000	93.96	0.670	0.001
Other disorders (any grade)	0.097 (0.092–0.103)	0.010 (0.006–0.018)	318.8	0.000	84.00	0.000	0.000
3–4 grade	0.022 (0.020–0.025)	0.022 (0.015–0.031)	202.6	0.000	74.82	0.049	0.930
*Renal disorders*							
Any grade	0.164 (0.145–0.185)	0.031 (0.019–0.050)	272.0	0.000	84.56	0.245	0.000
3–4 grade	0.011 (0.0078–0.016)	0.011 (0.007–0.016)	33.55	0.821	0.000	0.000	0.001

**Fig 1:**
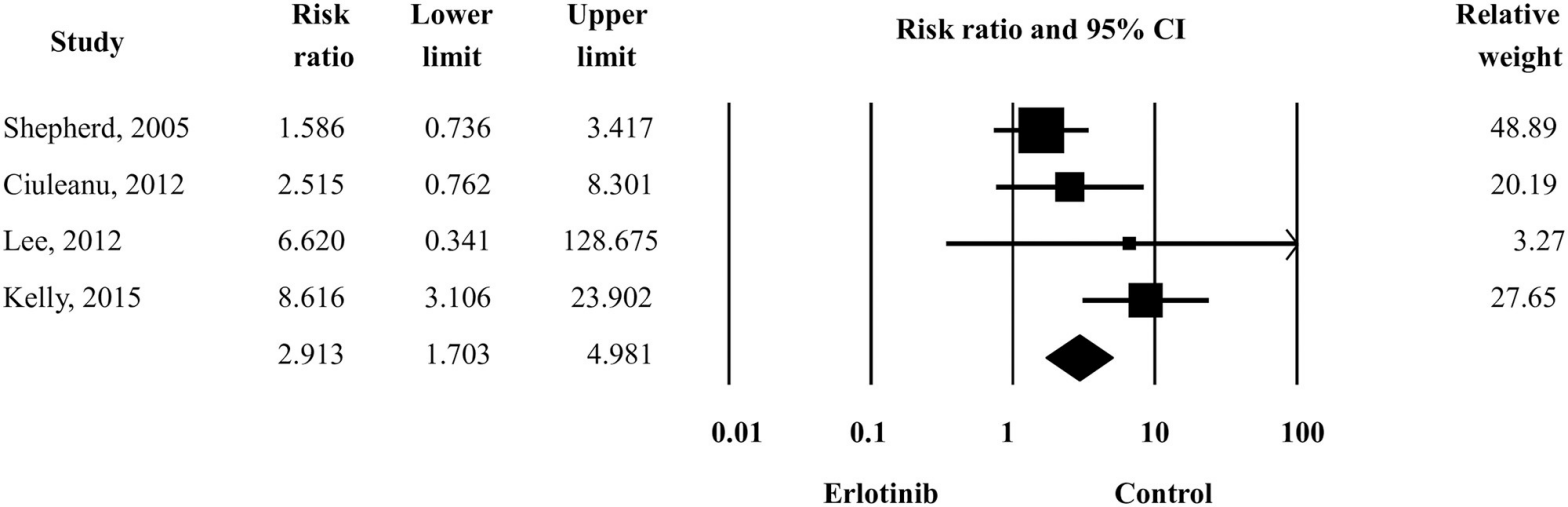
Forest plot of relative risk of eye disorders in the erlotinib treatment group *vs*. the control group.

Four studies were assessed for the specific contribution of erlotinib to the development of eye disorders by comparing erlotinib-treatment groups and control groups (placebo or other treatment). The risk ratios (RR) and 95% CI of the comparison between the two groups were 2.91 and 1.70–4.98, respectively (Fig 1). Reanalysis using a random-effects model revealed the significant differences (RR = 3.34; 95% CI 1.32–8.45). This result indicated that patients who received erlotinib had significantly increased the risk of ocular toxicities. Test of heterogeneity and publication bias for this comparison are presented in Table 3.

**Table 3:** Test of heterogeneity and publication bias in comparisons between the erlotinib-treatment group and control group.

Types of disorder	Heterogeneity			Publication bias	
	*Q v*alue	*P v*alue	*I*^2^	*P* value (Begg’s)	*P* value (Egger’s)
Eye disorders	7.102	0.069	57.76	0.743	0.582
ALT elevations	5.514	0.356	9.323	0.133	0.208

### Incidence and relative risk of erlotinib-induced hepatobiliary disorders

Fifty-two of the 60 studies were assessed for the incidence of hepatobiliary disorders in 21,339 participants treated with erlotinib (Table 1). The index of hepatobiliary disorders mainly included alanine transaminase (ALT) or aspartate transaminase (AST) elevations, alkaline phosphatase elevation, hyperbilirubinemia, and other hepatobiliary adverse events. In detail, 751 ALT increases, 456 bilirubin increases, and 1,025 other adverse events of any grade were reported and 301 of them were classified as grade 3–4. The incidence in each study of the ALT increase ranged from 0.00% to 50.9% and the incidence of bilirubin increase ranged from 0.00% to 38.9%.

The overall incidence (event rate) of ALT and bilirubin increases were 6.40% (95% CI 3.90–10.4) and 3.8% (95% CI 2.30%–6.10%), respectively, using the random-effects model (Table 2). The overall incidence of other adverse events except ALT and bilirubin increases were 1.00% (95% CI 0.60%–1.80%). The incidence of any hepatobiliary disorders of grade 3–4 was 2.20% (95% CI 1.50%–3.10%) (Table 2).

**Fig 2:**
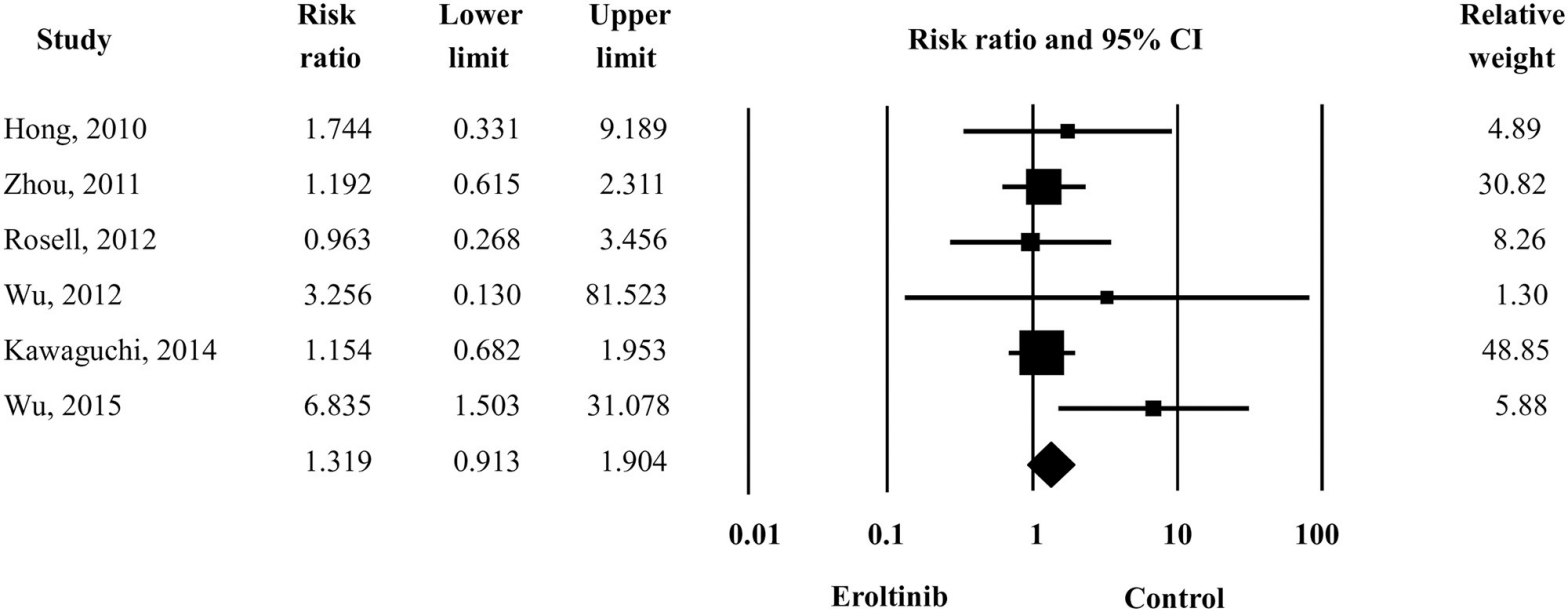
Forest plot of relative risk of ALT elevation in the erlotinib treatment group *vs*. the control group.

Five studies were included to compare the liver toxicity of erlotinib, representing as ALT elevation, between erlotinib-treatment groups and control groups (placebo or other treatment). The RR and 95% CI of the comparison between the two groups were 1.319 and 0.913–1.904, respectively, using the fixed-effects model (Fig 2). Reanalysis using a random-effects model revealed no significant differences. This result indicated that patients who received erlotinib had no significantly increased risk of liver-related toxicities. Test of heterogeneity and publication bias for this comparison are presented in Table 3.

### Incidence of erlotinib-induced renal disorders

Forty-three studies were assessed for erlotinib-induced renal disorders in 10,367 participants treated with erlotinib (Table 1). In total, 218 renal adverse events of any grade were reported and nine of the 218 adverse events were classified as grade 3–4. The incidence of renal disorders in each study ranged from 0.00% to 25.4%. The index of renal disorders mainly included elevated serum creatinine, proteinuria, renal failure, and other renal adverse events.

The overall incidence (event rate) of renal disorders was 3.10% (95% CI 1.90%–5.00%), using the random-effects model (Table 2). The incidence of renal disorder of grade 3–4 was 1.10% (95% CI 0.70%–1.60%), using the fixed-effects model (Table 2). Reanalysis using a random-effects model showed the same result.

### Sensitivity analysis and publication bias

As the results of sensitivity analysis, no significant differences were observed (data available on request). The results of publication bias through the Begg’s rank correlation test and Egger’s regression test are shown in Tables 2 and 3.

## Discussion

This meta-analysis was conducted to evaluate the eye, hepatobiliary, and renal toxicities of erlotinib in patients with EGFR positive NSCLC. The present meta-analysis quantitatively integrated the inconsistent results of reported clinical studies.

In the present meta-analysis, phase I studies were not included due to divergence from the dosage regimens in phase II, III, and IV studies. The reason is that the dose regimen of erlotinib is a highly associating factor in assessing the toxicity of erlotinib. The most common toxicities associated with erlotinib in phase I studies were also dose-dependent rash and diarrhea which were similarly observed in the included phase II, III, or IV studies [74, 75]. Other reported adverse events included mucositis, nausea, vomiting, and headaches that were observed less frequently [74].

Warnings and precautions were described as pulmonary toxicity, renal failure, hepatotoxicity, cardiovascular events, and ocular disorders, according to the approved drug information for erlotinib. Those adverse events were less common, but mostly severe. The bigger issue is that the information is still not enough to refer for effective monitoring and study. Thus, the present meta-analysis is a meaningful and useful approach for erlotinib therapy.

Firstly, in the meta-analysis of the incidence of erlotinib-induced ocular disorders, the overall incidence was 3.30% and the incidence of grade 3–4 disorders was 0.40% in patients with NSCLC cancers (Table 2). Comparing to the control groups, the risk of eye toxicities was significantly higher in the erlotinib group (Fig 1). EGFR is present in the eyes (corneal and conjunctival epithelial cells) and is also expressed in the sebaceous glands and hair follicle sheaths [76, 77]. EGFR in the above tissue plays an important role in regulating cell proliferation, apoptosis, and differentiation [78]. Whereas, erlotinib, as an EGFR inhibitor, interferes with the regulatory mechanism of EGFR and the eye toxicity is thought to be linked to the EGFR inhibition [6, 79, 80]. Erlotinib-induced eye disorders can be relieved by discontinuation of the treatment, but some cases may be more severe and manifest as advanced or irreversible diseases. Thus, regular follow-up relating to ocular disorders should be considered in all patients treated with erlotinib for prevention, early diagnosis, and treatment.

Secondly, in the evaluation of the incidence of erlotinib-induced hepatobiliary disorders, the overall incidence of ALT and bilirubin elevations were 6.40% and 3.80%, respectively. But, the risk of hepatobiliary toxicities was not significantly increased in the erlotinib group, compared to the control group. It was reported that liver function test abnormalities were common (1% to 10%) adverse events in the post-marketing data with over 400,000 patients with NSCLC having received erlotinib [81]. The events were mainly mild to moderate, transient, or associated with liver metastasis. Both the present meta-analysis and post-marketing data similarly indicate that ALT or bilirubin elevations are frequently observed, but not critical toxicity necessary to factor into treatments with erlotinib. However, the erlotinib-induced hepatotoxicity could be increased by other risks or features patients may have. A recent retrospective study showed that concomitant use of CYP3A4 inducers and H2-antagonist/PPIs, liver metastasis, and age ≥65 were risk factors of erlotinib-induced hepatotoxicity [82]. Therefore, a monitoring strategy for hepatobiliary toxicities of erlotinib should be recommended persistently in patients with these risk factors mentioned above.

Lastly, the overall incidence of renal disorder was evaluated. Kidney-related toxicity of erlotinib has not been extensively researched because erlotinib is mainly metabolized by CYP3A4, CYP1A1, and CYP1A2, in the liver. Safety concerns for patients with renal failure have rarely been reported, but one pharmacokinetic study showed that erlotinib was hardly affected by renal function and hemodialysis in patients with NSCLC and chronic renal failure [83]. Regarding this, a laboratory study has reported interesting results that erlotinib preserved renal function and prevented salt retention in nephrotic rats [84]. Another laboratory study has reported similar conclusions that erlotinib attenuated the progression of chronic kidney disease in rats with remnant kidney [85]. Further clinical studies will hopefully provide answers about this issue.

Interestingly, the antidiabetic effects of TKIs are reported from several clinical and non-clinical researches and are also suggested as a new approach for diabetes mellitus [86]. This may be explained that TKIs indirectly improves insulin sensitivity by inhibiting EGFR. Furthermore, one recent study has indicated that inhibition of EGFR by erlotinib is associated with improved diabetic nephropathy and insulin resistance in animal model with type 2 diabetes [87]. The above results are also associated with the low risk of renal disorder in the present meta-analysis.

The inevitable limitation of meta-analysis is that analysis should be performed based on previously reported studies and those studies are not necessarily complete or accurate. Likewise, there was a lack of original data in the present meta-analysis, and the studies differed substantially with regard to dosage regimens and study periods. Despite these limitations, the strength of current meta-analysis is that more than 1,500 articles were reviewed and a sufficient number of studies were included in the analysis.

## Conclusions

We examined the overall incidence of the erlotinib-induced eye, hepatobiliary, and renal disorders in patients with NSCLC. Based on the results, careful monitoring of eye toxicity in patients receiving erlotinib should be recommended and close monitoring including liver function tests should be suggested in patients with hepatic toxicity-related risk factors.

## Supporting information

S1 Fig PRISMA diagram of the process of selecting relevant studies. (DOCX)

S1 Checklist PRISMA 2009 checklist. (DOC)
