# Supplementary material for: Eye, hepatobiliary, and renal disorders of erlotinib in patients with non-small-cell lung cancer: A meta-analysis
Source: PLoS One. 2020 Jul 14;15(7):e0234818. doi: 10.1371/journal.pone.0234818 (PMC7360022; doi:10.1371/journal.pone.0234818)
Supplement: S1 Fig — (DOCX) [file pone.0234818.s001.docx]

**Supplementary figure**

**Fig. 1.** PRISMA diagram of the process of selecting relevant studies

Potentially relevant articles identified and screened

(n = 1,511)

Articles after duplicates removed

(n = 1,148)

Full-text articles assessed for eligibility

(n = 214)

Articles excluded after title and abstract screening with inclusion criteria

(n = 934)

Articles included in quality assessment

(n = 60)

Articles excluded based on full text

(n = 154)

- 20, Insufficient data
- 2, Overlapping data
- 132, Unsuitable based on the inclusion criteria

Articles retrieved for more detailed evaluation

- Eye-disorder (n = 44)
- Hepatobiliary disorder (n = 52)
- Renal disorder (n = 43)
